# Correction: Kaushal et al. Repurposing Niclosamide for Targeting Pancreatic Cancer by Inhibiting Hh/Gli Non-Canonical Axis of Gsk3β. *Cancers* 2021, *13*, 3105

**DOI:** 10.3390/cancers13225591

**Published:** 2021-11-09

**Authors:** Jyoti B. Kaushal, Rakesh Bhatia, Ranjana K. Kanchan, Pratima Raut, Surya Mallapragada, Quan P. Ly, Surinder K. Batra, Satyanarayana Rachagani

**Affiliations:** 1 Department of Biochemistry and Molecular Biology, University of Nebraska Medical Center, Omaha, NE 68198, USA; jyoti.kaushal@unmc.edu (J.B.K.); rocky.bhatia@unmc.edu (R.B.); ranjana.kanchan@unmc.edu (R.K.K.); pratima.raut@unmc.edu (P.R.); sbatra@unmc.edu (S.K.B.); 2 Department of Chemical and Biological Engineering, Nanovaccine Institute, Iowa State University, Ames, IA 50011, USA; suryakm@iastate.edu; 3 Department of Surgical Oncology, University of Nebraska Medical Center, Omaha, NE 68198, USA; qly@unmc.edu; 4 Fred & Pamela Buffet Cancer Center, Eppley Institute for Research in Cancer and Allied Diseases, University of Nebraska Medical Center, Omaha, NE 68198, USA

The authors would like to make a correction to their published paper [1].

On page 13, in the online version of this article, the “fluorescence image in Figure 4D″ was mistakenly misplaced with a fluorescence image of Figure 2C during submission/revision. The authors would like to change Figure 4 with a new corrected version (with replaced Figure 4D), which is listed below:

The change does not affect the scientific results.

The rest of the manuscript does not need to be changed. The authors would like to apologize for any inconvenience caused. The original manuscript has been updated.

## Figures and Tables

**Figure 4:**
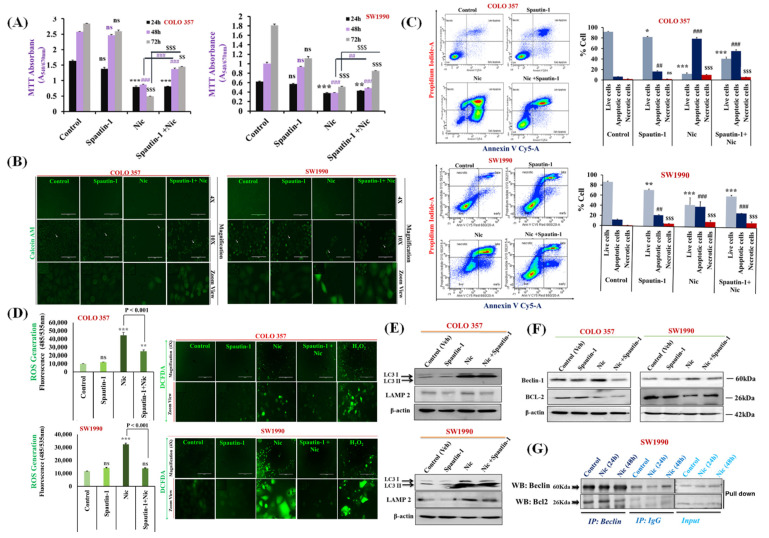
Nic-induced autophagy leads to PC cell death by disrupting Beclin1–BCL2 interaction. (**A**) Effect of functional blockage of autophagy via spautin-1 on Nic-treated PC cells resulted in growth promotion. COLO 357 and SW1990 cells were pretreated with spautin-1 for 2 h, followed by Nic (10 µM) for 24, 48, and 72 h and cell viability was assessed by MTT assay. Values are expressed as mean ± SEM (*n* = 3), *p* values: *** *p* < 0.001, ** *p* < 0.01 vs. control (24 h); *p* values: **^###^** *p* < 0.001, **^##^** *p* < 0.001 vs. control (48 h); *p* values: ^$$$^ *p* < 0.001, ^$$^ *p* < 0.001 vs. control (72 h). (**B**) Representative images showing the Calcein-AM-stained cells, original magnification 4×; 10×. (**C**) Protective effect of autophagy inhibition on Nic-induced apoptosis was determined by flow cytometric analysis of PC cell lines after staining with annexin-V Cy-5/PI (AV^+^/PI—intact cells; AV/PI^+^—nonviable/necrotic cells; AV^+^/PI and AV^+^/PI^+^—apoptotic cells) in different groups, i.e., spautin-1, Nic and combination of Spautin-1 and Nic (left panel). Quantitative analysis of these micrographs was shown as mean ± SEM, *p* values: *** *p* < 0.001, ** *p* < 0.01, * *p* < 0.05 vs. live control cells; **^###^** *p* < 0.001, **^##^** *p* < 0.001 vs. apoptotic control cells; ^$$$^ *p* < 0.001 vs. necrotic cells (right panel). (**D**) Intracellular ROS levels in cells treated with spautin-1, Nic and their combination were evaluated using DCFDA fluorogenic dye (10 µM for 30 min) by measuring fluorescence at 485/535 nm (excitation/emission) using a fluorescent plate reader (left panel). The results are expressed as relative fluorescence intensity units. Values are expressed as mean ± SEM (*n* = 3), *p* values: *** *p* < 0.001, ** *p* < 0.01 vs. control. Fluorescence imaging was performed in these cells stained with DCFDA (right panel), original magnification 4×. (**E**,**F**) Representative western blot images showing the expression of LC3I/II and LAMP2 (**E**); Beclin-1 and BCL2 (**F**) in COLO 357 and SW1990 cells treated with spautin-1, Nic and their combination. Membrane was stripped and re-probed with β-actin to correct for loading control. (**G**) Representative western blot images showing the expression of Beclin-1 and BCL2 in cells treated with Nic (10 µM; 24 and 48 h) and lysates were immunoprecipitated with Beclin-1 antibody and analyzed by western blotting for BCL2 interaction.
