# Supplementary material for: Repurposing Niclosamide for Targeting Pancreatic Cancer by Inhibiting Hh/Gli Non-Canonical Axis of Gsk3β
Source: Cancers (Basel). 2021 Jun 22;13(13):3105. doi: 10.3390/cancers13133105 (PMC8269055; doi:10.3390/cancers13133105)
Supplement: Supplementary file 1 [file cancers-13-03105-s001.zip › cancers-1215735-supplementary.pdf]

cancers

MDPI

Article

Repurposing Niclosamide for Targeting Pancreatic Cancer by Inhibiting Hh/Gli Non-Canonical Axis of Gsk3β

Jyoti B. Kaushal, Rakesh Bhatia, Ranjana K. Kanchan, Pratima Raut, Surya Mallapragada, Quan P. Ly, Surinder K. Batra and Satyanarayana Rachagani

Supplementary Materials:

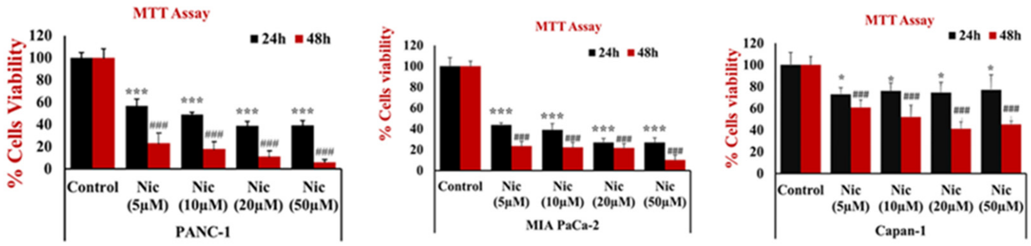

Figure S1. Inhibitory effect of Nic on poorly and well differentiated PC cell lines. Concentration (1, 5, 10 and 20 µM) and time-dependent (24 and 48 h) effect of Nic treatment on viability of poorly differentiated (PANC-1, MIAPaCa-2) and well differentiated (Capan-1) pancreatic cell lines was determined by MTT assay. Values are expressed as mean ± SEM (n=6), p values: ***$p < 0.001$, *$p < 0.05$ vs control (24 h); ###$p < 0.001$ vs control (48 h).

Citation: Kaushal, J.B.; Bhatia, R.; Kanchan, R.K.; Raut, P.; Mallapragada, S.; Ly, Q.P.; Batra, S.K.; Rachagani, S. Repurposing Niclosamide for Targeting Pancreatic Cancer by Inhibiting Hh/Gli Non-Canonical Axis of Gsk3β. *Cancers* **2021**, *13*, 3105. https://doi.org/10.3390/cancers13133105

Academic Editor: Murray Korc

Received: 24 April 2021
Accepted: 17 June 2021
Published: 22 June 2021

Publisher's Note: MDPI stays neutral with regard to jurisdictional claims in published maps and institutional affiliations.

Copyright: © 2021 by the authors. Licensee MDPI, Basel, Switzerland. This article is an open access article distributed under the terms and conditions of the Creative Commons Attribution (CC BY) license (http://creativecommons.org/licenses/by/4.0/).

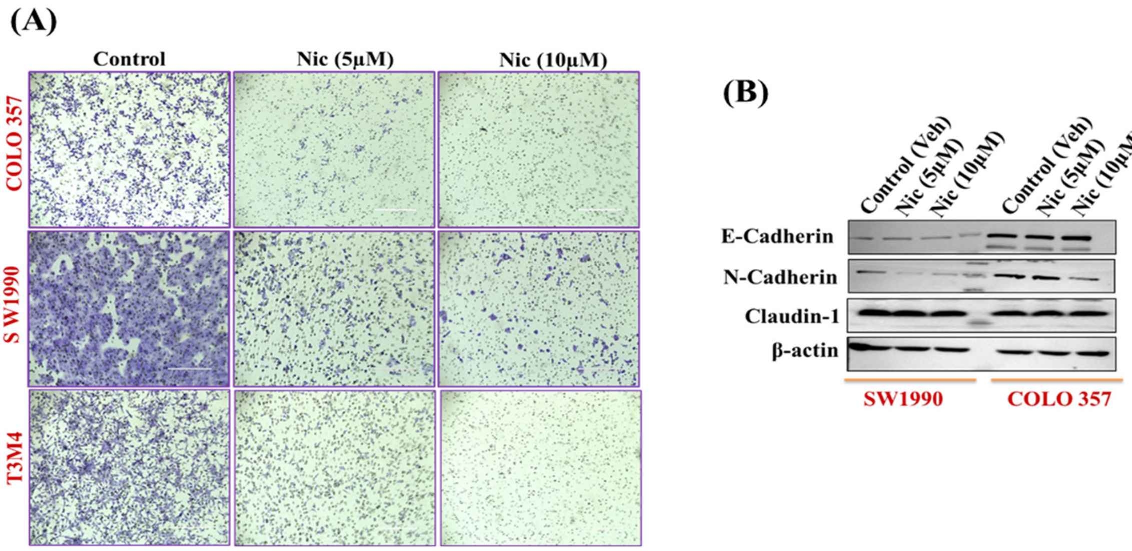

Figure S2. Nic-mediated inhibition of migration potential in PC cell lines. (**A**) Nic-treated COLO 357, SW1990, and T3M4 cells were analyzed for their migration potential. A total of $1{\times}10^5$ cells were seeded into top chamber of the trans-well with serum-free media, whereas, bottom chamber with serum containing media, and after 24 h, migrated cells were stained and analyzed. (**B**) Representative western blots showing the expression of protein markers involved in migration and EMT transition such as E-cadherin, N-cadherin, claudin in PC cell lines (COLO 357 and SW1990) upon treated with Nic (5µM and 10µM).

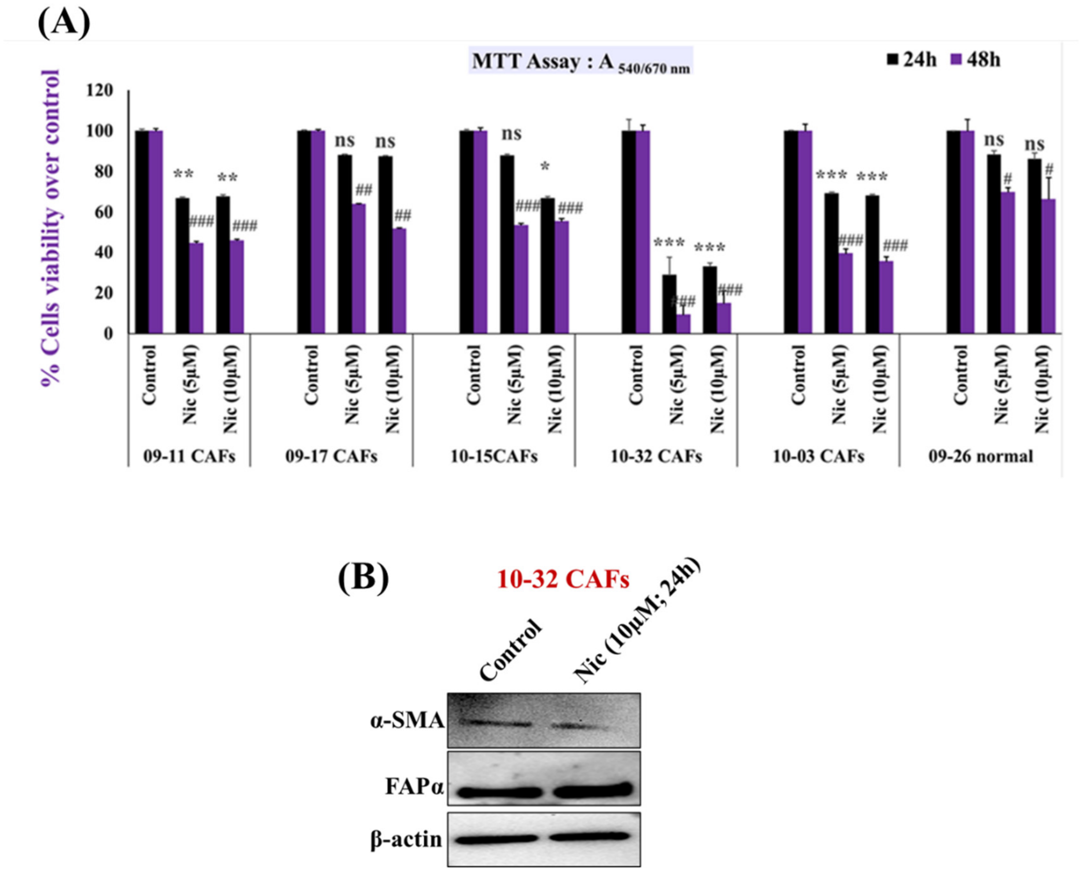

Figure S3. The efficacy of Nic on pancreatic cancer-associated fibroblasts. (**A**) The effect of Nic on viability of cancer-associated fibroblast cell lines (09-11, 09-17, 10-32, 10-15, 10-03CAFs) and normal fibroblast (09-26N) were determined by MTT assay. Values are expressed as mean ± SEM(n=6), p values: ***$p < 0.001$, **$p < 0.001$, *$p < 0.05$ vs control (24 h); ###$p < 0.00$, ##$p < 0.001$, #$p < 0.05$ vs control (48 h) (**B**) Representative western blot image showing the expression of α-SMA and FAPα in 10-32 CAFs cells treated with Nic (10 µM) for 24 h.

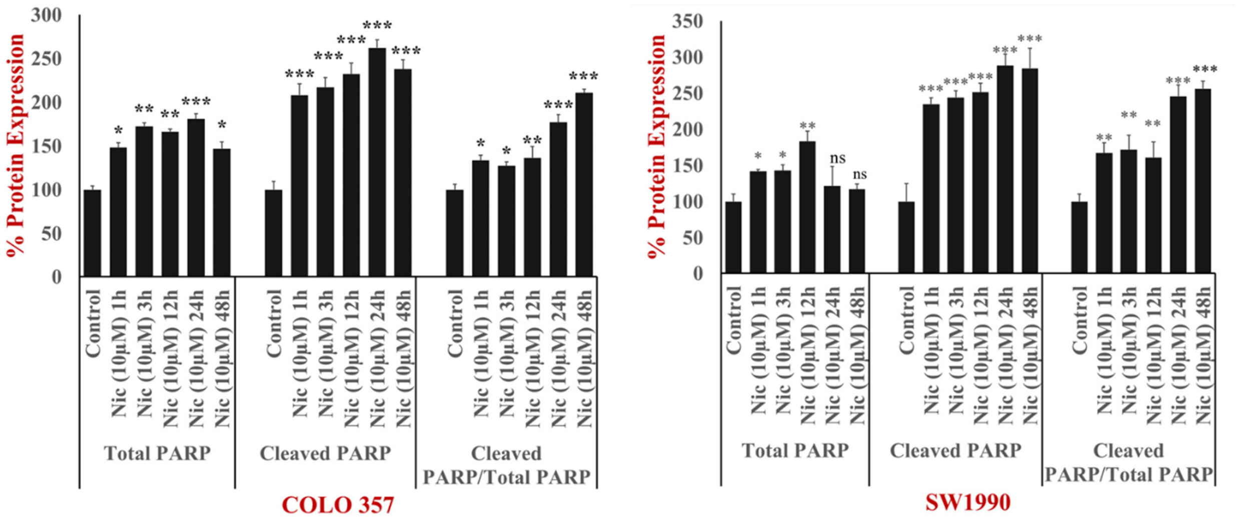

Figure S4. Analysis of PARP expression in PC cell lines upon Nic treatment. Densitometric quantitation of western blot results shown as percentage protein expression of full-length PARP, cleaved PARP, and the ratio of cleaved to full-length PARP in COLO 357 and SW1990 cells treated with Nic (10 µM) for various time periods i.e., 1, 3, 12, 24, and 48 h. Values are expressed as mean ± SEM (n = 3), p values: ***$p < 0.001$, **$p < 0.001$, *$p < 0.05$, $^{ns}p > 0.05$ vs control.

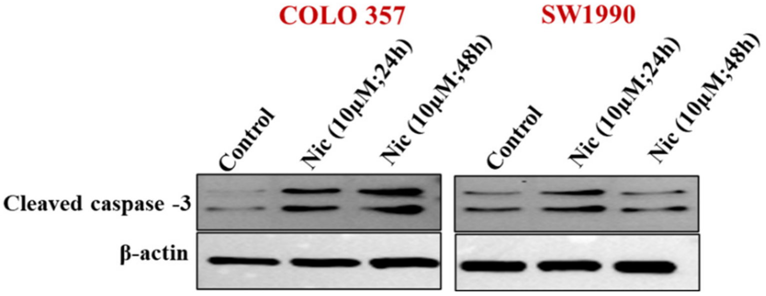

Figure S5. Effect of Nic on the Cleaved caspase-3 expression in PC cell lines. Representative western blot images showing the expression of Cleaved caspase-3 in COLO 357 and SW1990 PC cell lines treated with Nic (10 µM) for 24 h and 48 h and observed time dependent increase of cleaved caspase-3 expression in PC cell lines.

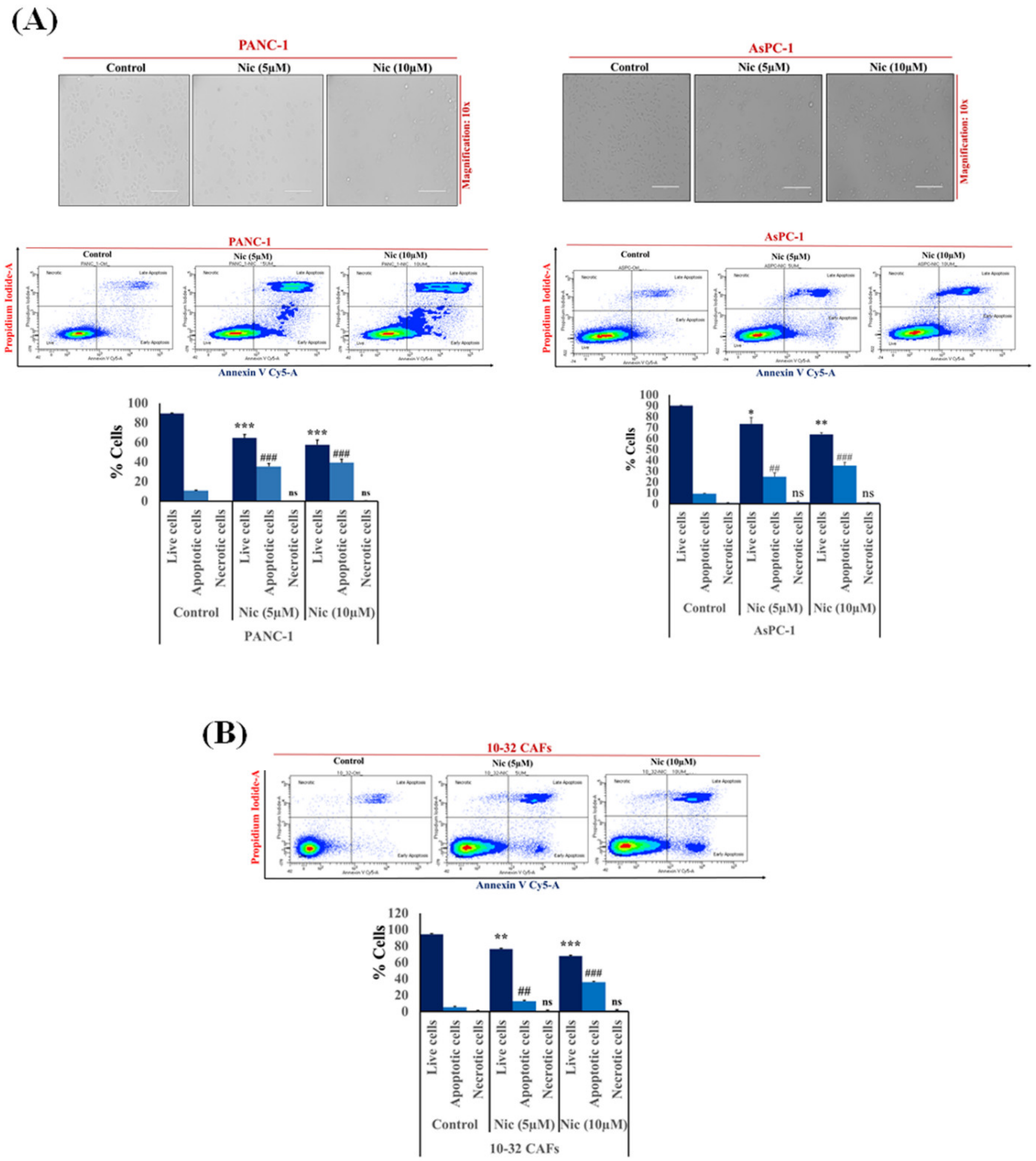

Figure S6. Nic exhibits growth suppression in poorly differentiated PC cell lines and pancreatic cancer-associated fibroblasts (CAFs) by inducing apoptosis. (**A**) Poorly differentiated PC cell lines (PANC-1 and AsPC-1) and (**B**) pancreatic cancer associated fibroblast cell line (10-32 CAFs) were treated with Nic (5 and 10 µM) for 24 h, and induction of apoptosis was determined by flow cytometric analysis of annexin-V Cy-5/PI- dual stained cells (AV+/PI–intact cells; AV/PI+–nonviable/necrotic cells; AV+/PI and AV+/PI+ – apoptotic cells) in PC cell lines (left panel). Quantitative analysis of these micrographs was shown as mean ± SEM (n = 3), *p* values: ***$p < 0.001$, **$p < 0.01$ vs live control cells; ###$p < 0.001$vs apoptotic control cells (right panel).

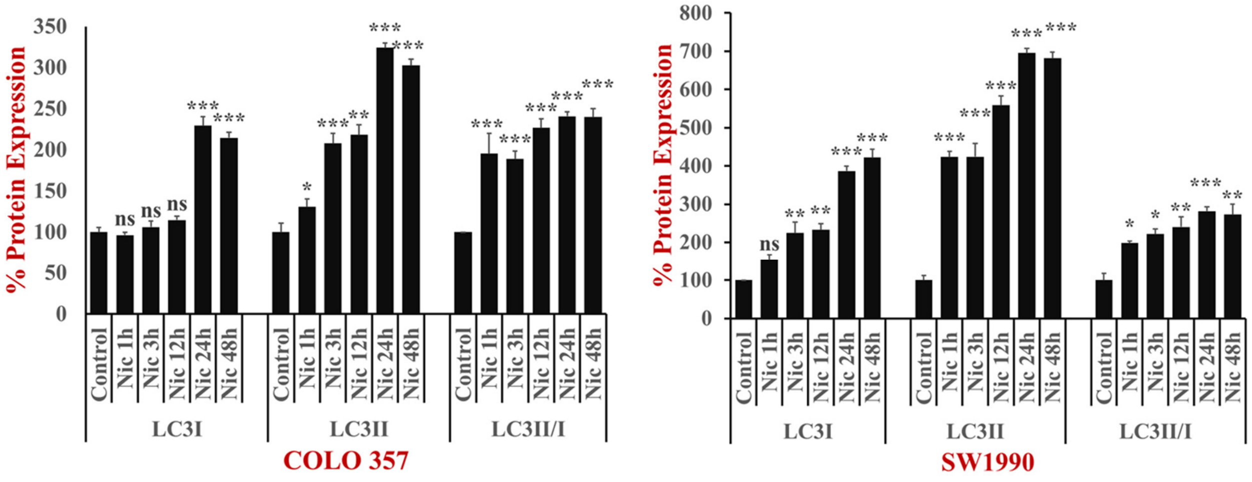

Figure S7. Nic stimulates the expression of a lipidated form of LC3B in PC cell lines. Densitometric quantitation of western blotting images was shown as percentage protein expression of LC3I, LC3 II, and LCII/I in COLO 357 and SW1990 cells treated with Nic (10 µM) for various time periods i.e., 1, 3, 12, 24, and 48 h. Values are expressed as mean ± SEM(n=3), p values: ***p < 0.001, **p < 0.001, *p < 0.05, ns$p$ >0.05 vs control.

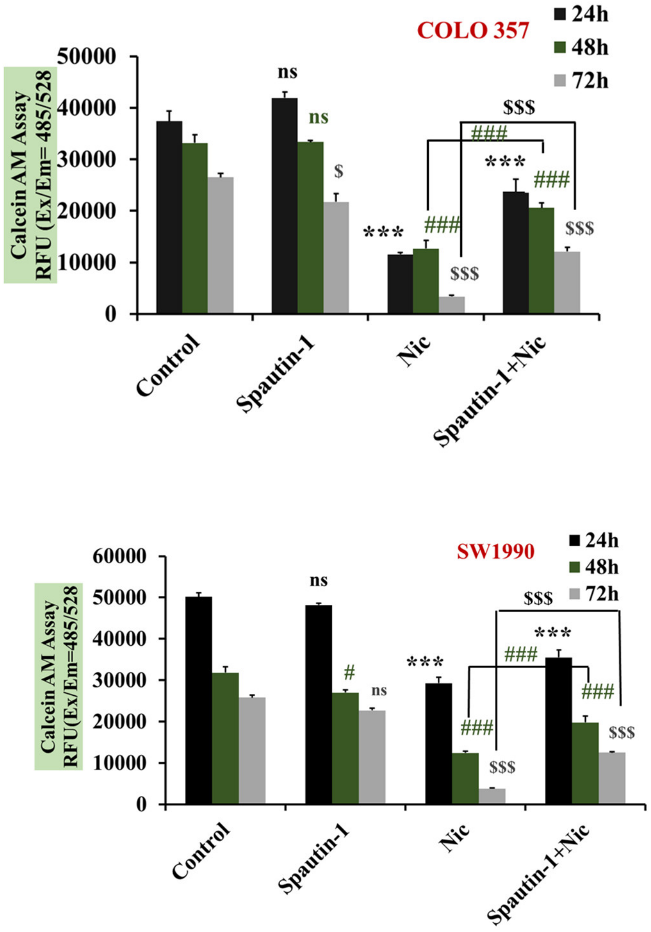

Figure S8. Effect of functional blockage autophagy via spautin-1 on Nic- mediated growth suppression via Calcein AM assay**.** COLO 357 and SW1990 cells were pretreated with spautin-1 for 2 h, followed by Nic (10 µM) for 24, 48, and 72 h and cell viability was assessed by Calcein-AM assay Values are expressed as mean ± SEM (n = 3), p values: ***p < 0.001, ***p < 0.001, *p < 0.05 vs control (24 h); p values: ###p < 0.001, ##p < 0.001, #p < 0.05 vs control (48 h); p values: \$\$\$$p$ < 0.001, \$\$$p$ < 0.001, \$$p$ < 0.05 vs control (72 h).

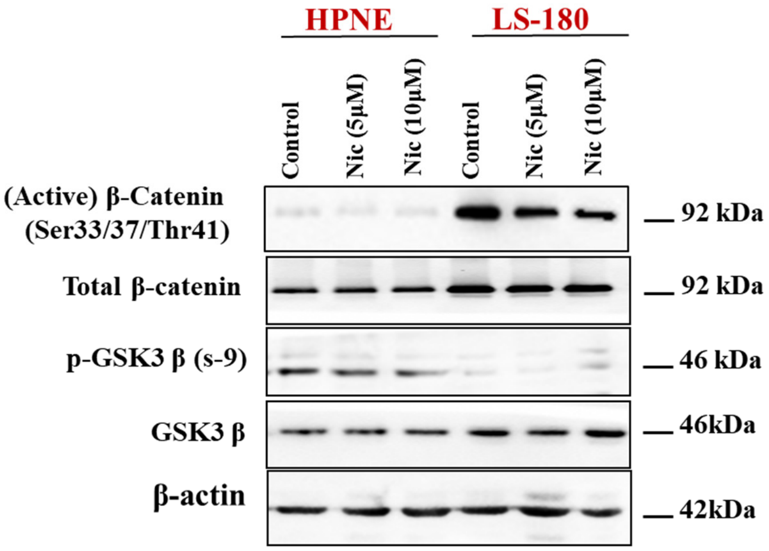

Figure S9. Demonstration of the effect of Nic on the β-catenin signaling molecules in normal pancreatic cell line (HPNE) and colorectal cancer cell line (LS-180). Representative western blot images showing the expression of β-catenin (active), p-Gsk3β, and Gsk3β in normal pancreatic cell line HPNE and colorectal cell line LS180 -treated with Nic (5 µM or10 µM) for 24 h. Membrane was stripped and reprobed with β-actin for internal loading control.

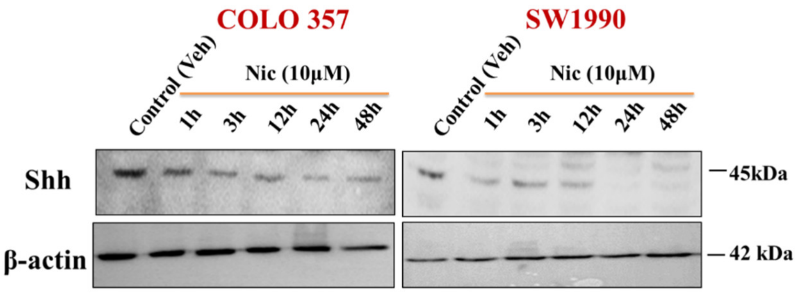

Figure 10. Demonstration of the effect of Nic on the expression of Hh signaling ligand (Shh) in PC cell lines. Representative western blot images showing the expression of Shh in PC cells treated with Nic (10 µM) for different time points. β-actin used as an internal loading control.

Figure 2

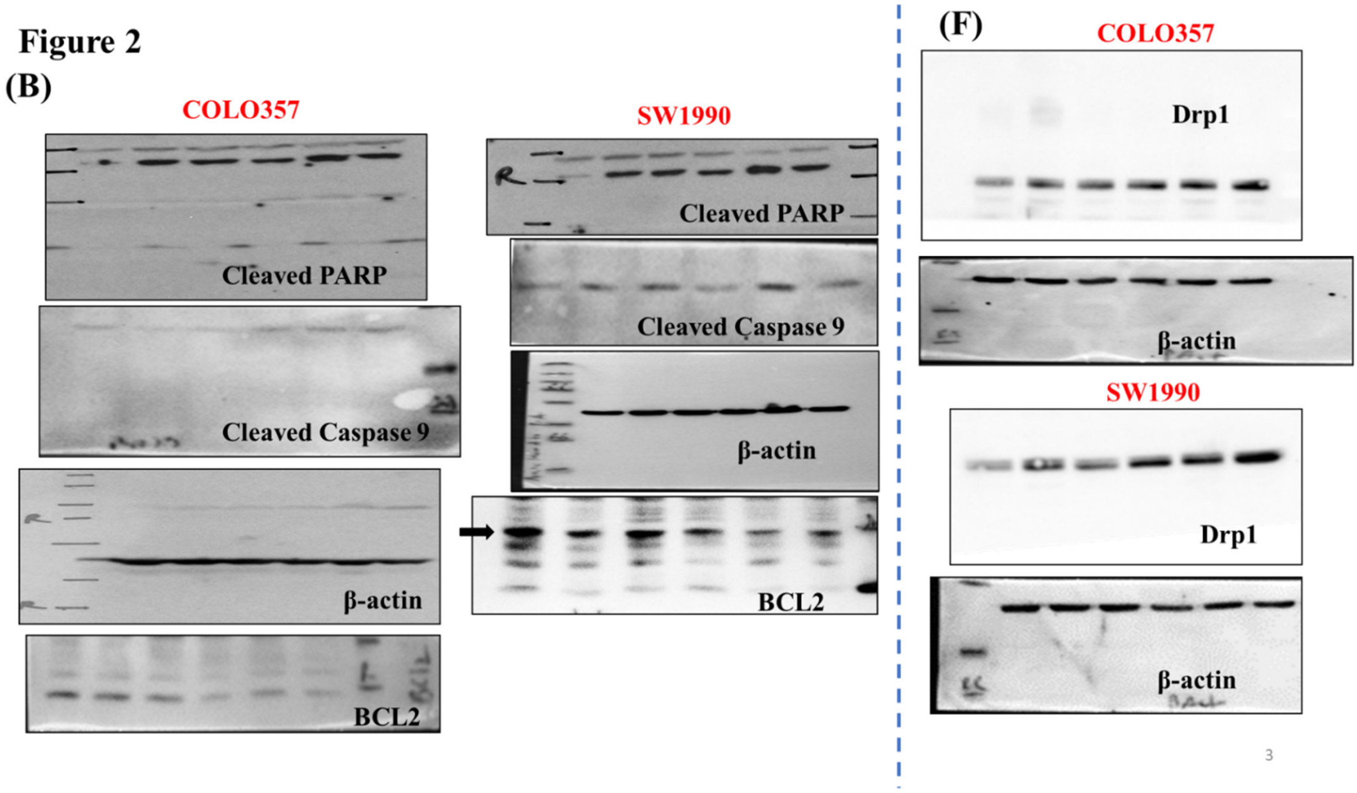

Figure 3

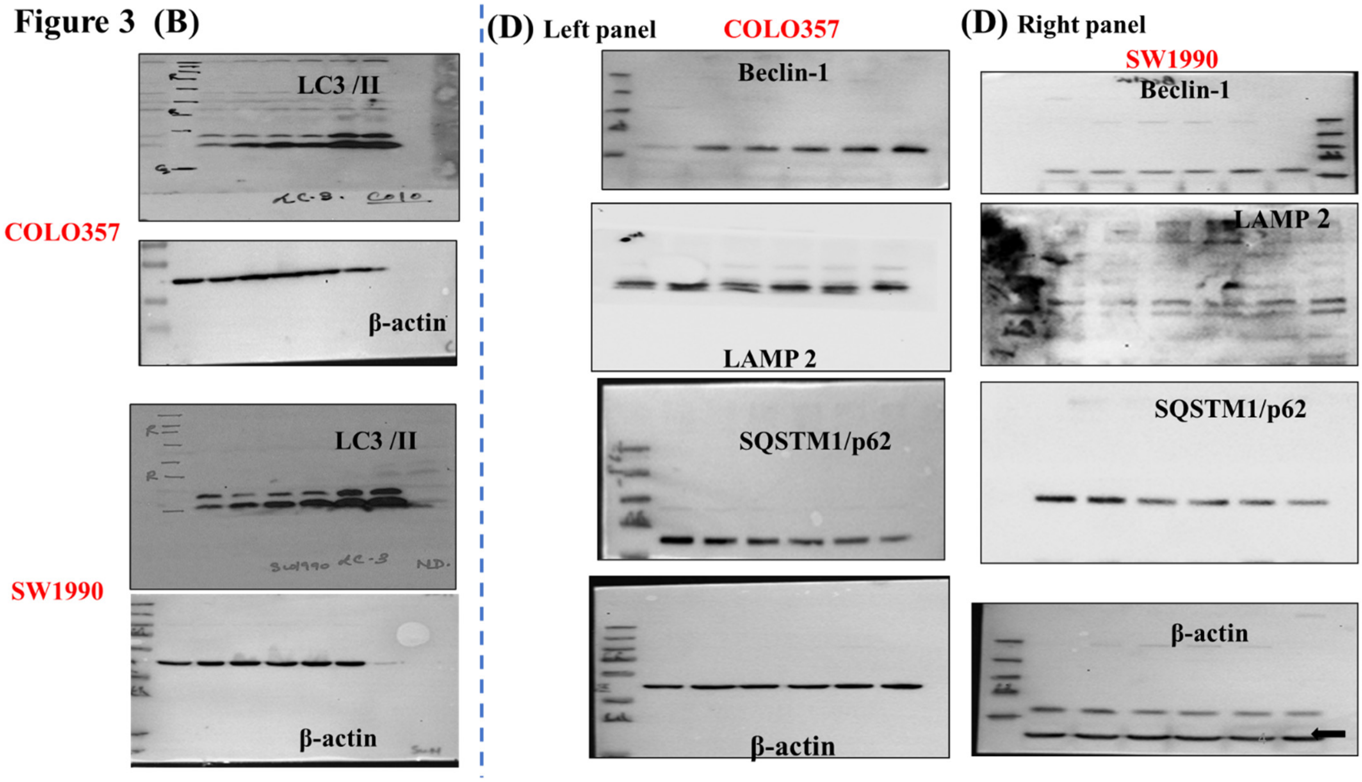

Figure 3

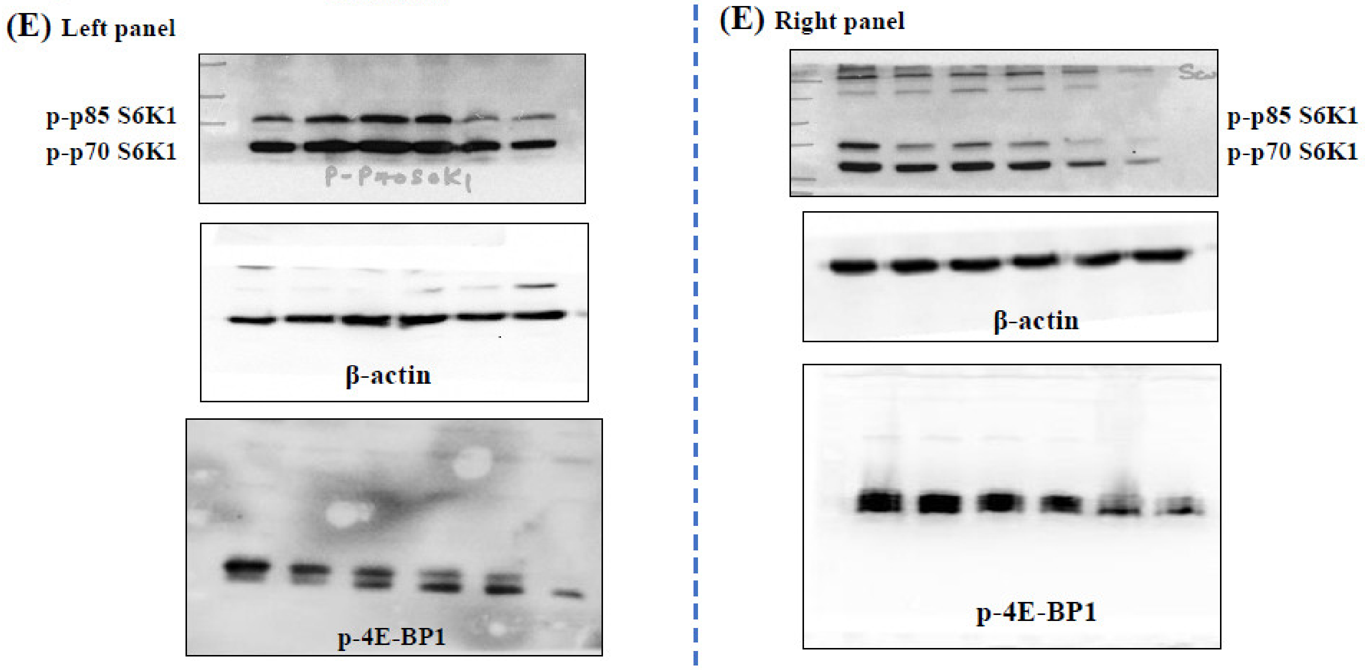

5

Figure 4

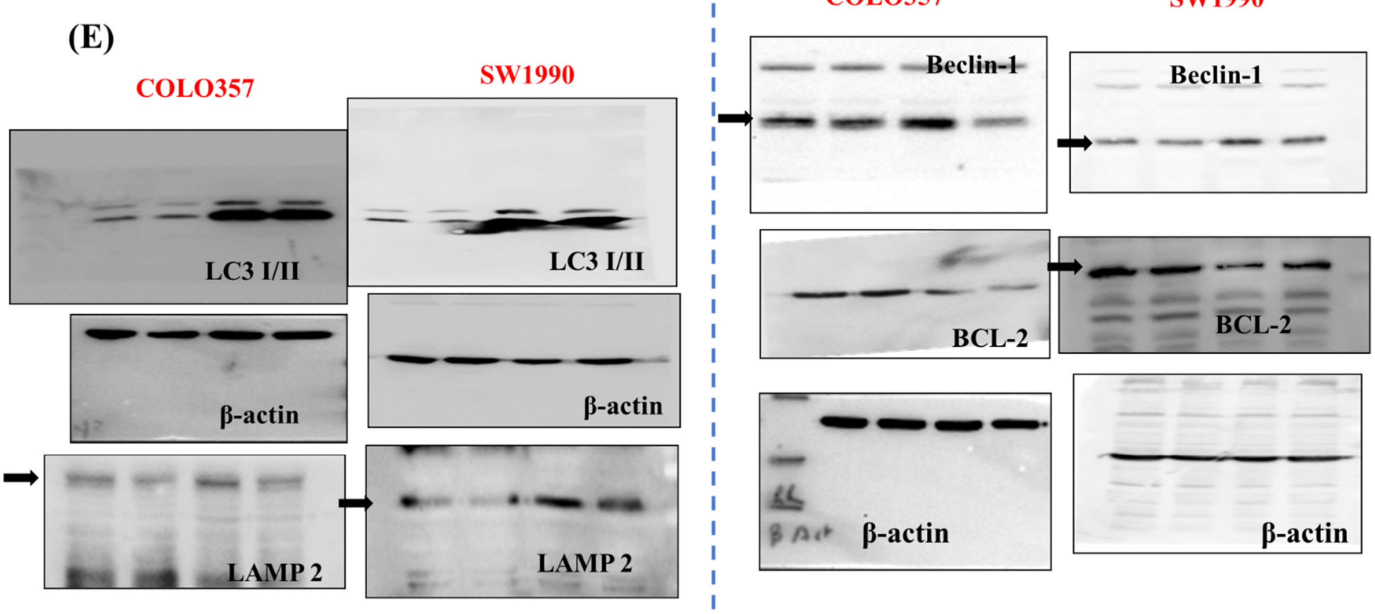

6

Figure 4 (G)

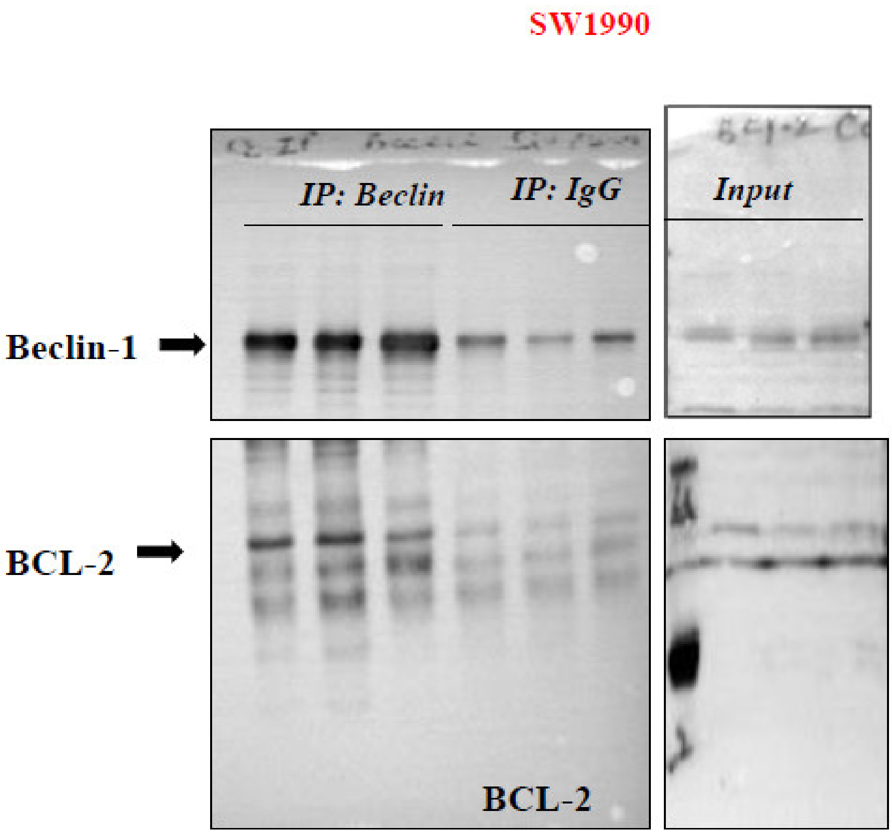

Figure 5 (A)

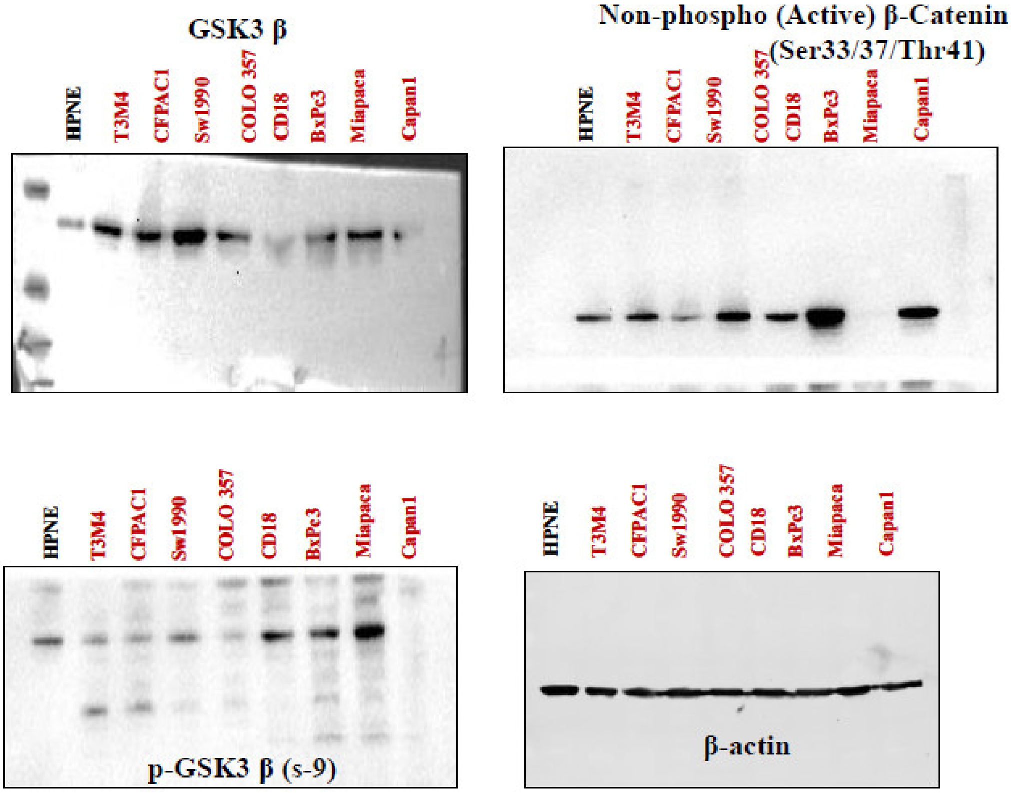

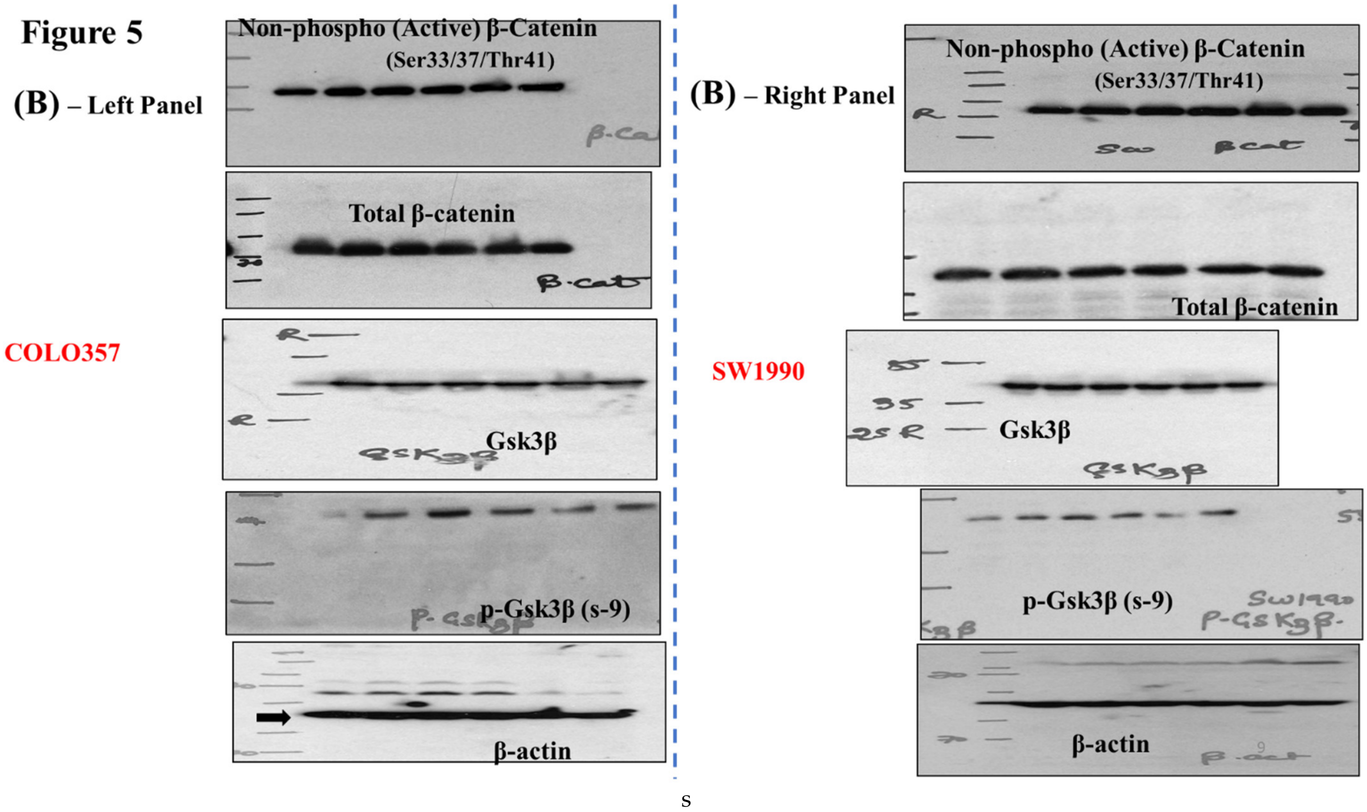
Figure 5
(B) – Left Panel
Non-phospho (Active) β-Catenin
(Ser33/37/Thr41)
Total β-catenin
COLO357
Gsk3β
p-Gsk3β (s-9)
β-actin
(B) – Right Panel
Non-phospho (Active) β-Catenin
(Ser33/37/Thr41)
Total β-catenin
SW1990
Gsk3β
p-Gsk3β (s-9)
β-actin

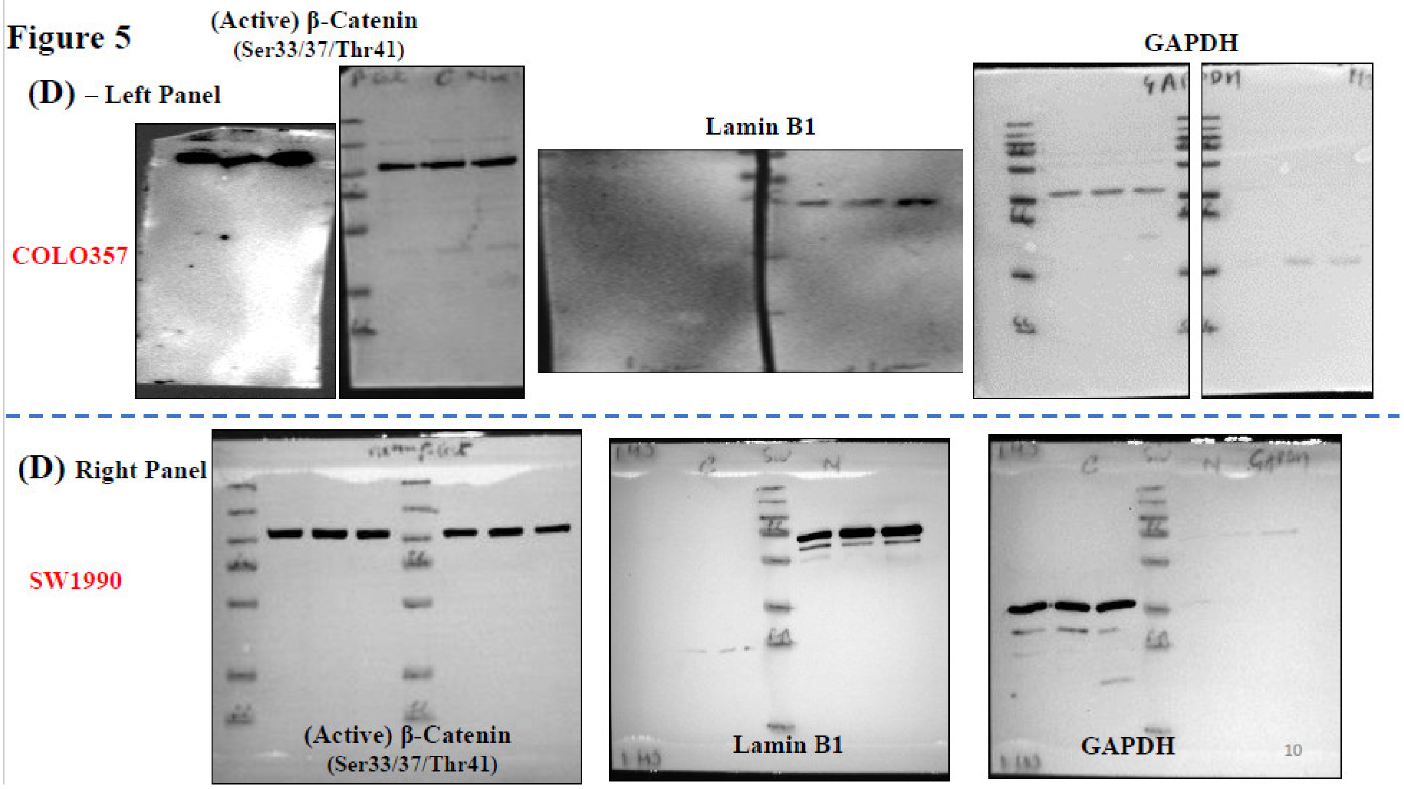
Figure 5
(D) – Left Panel
(Active) β-Catenin
(Ser33/37/Thr41)
COLO357
Lamin B1
GAPDH
(D) Right Panel
SW1990
(Active) β-Catenin
(Ser33/37/Thr41)
Lamin B1
GAPDH

Figure 6 (A)

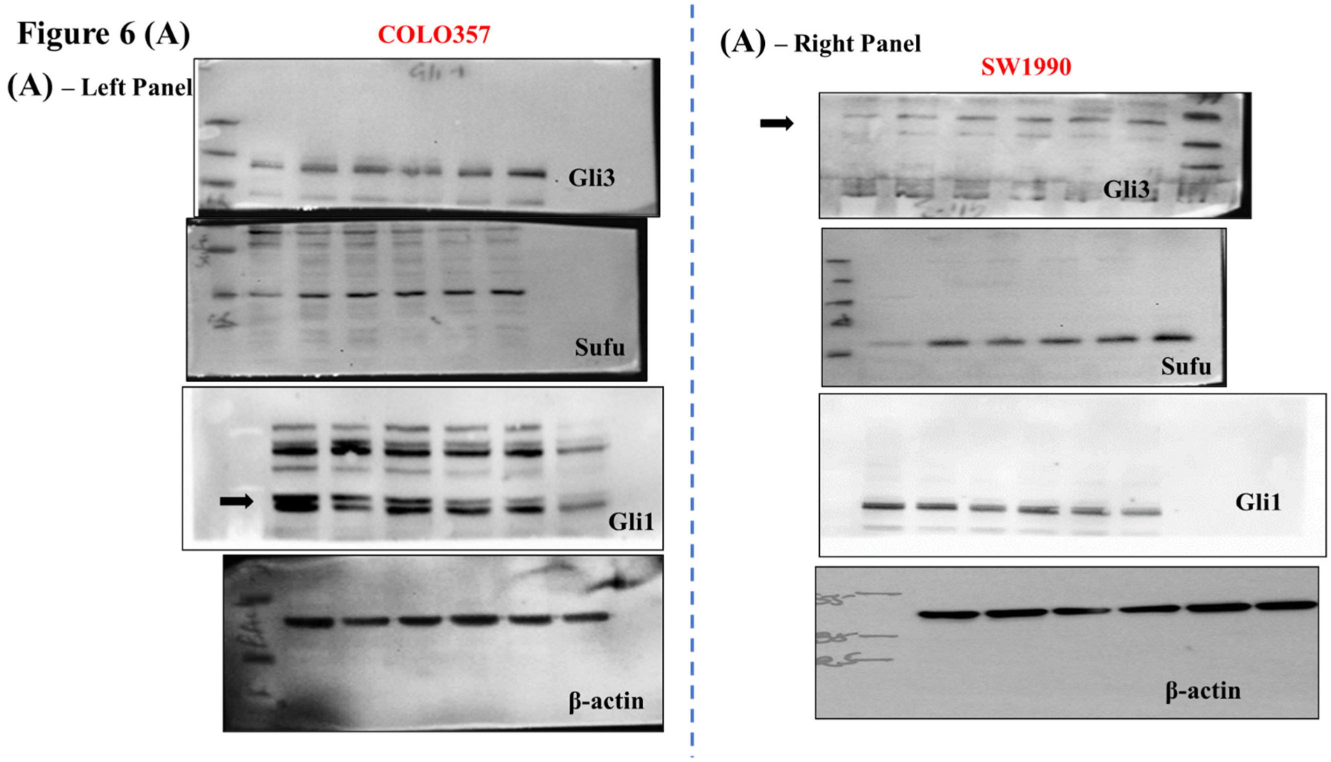

Figure 6 (B)

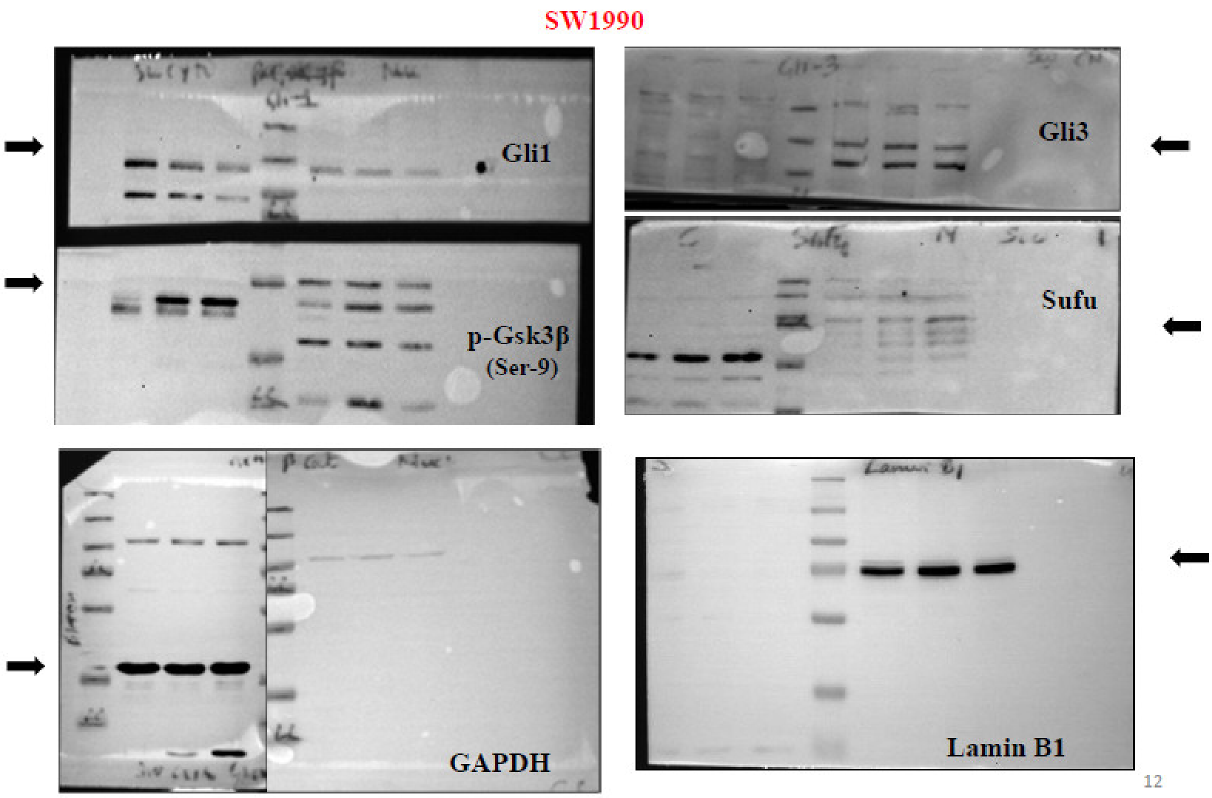

12

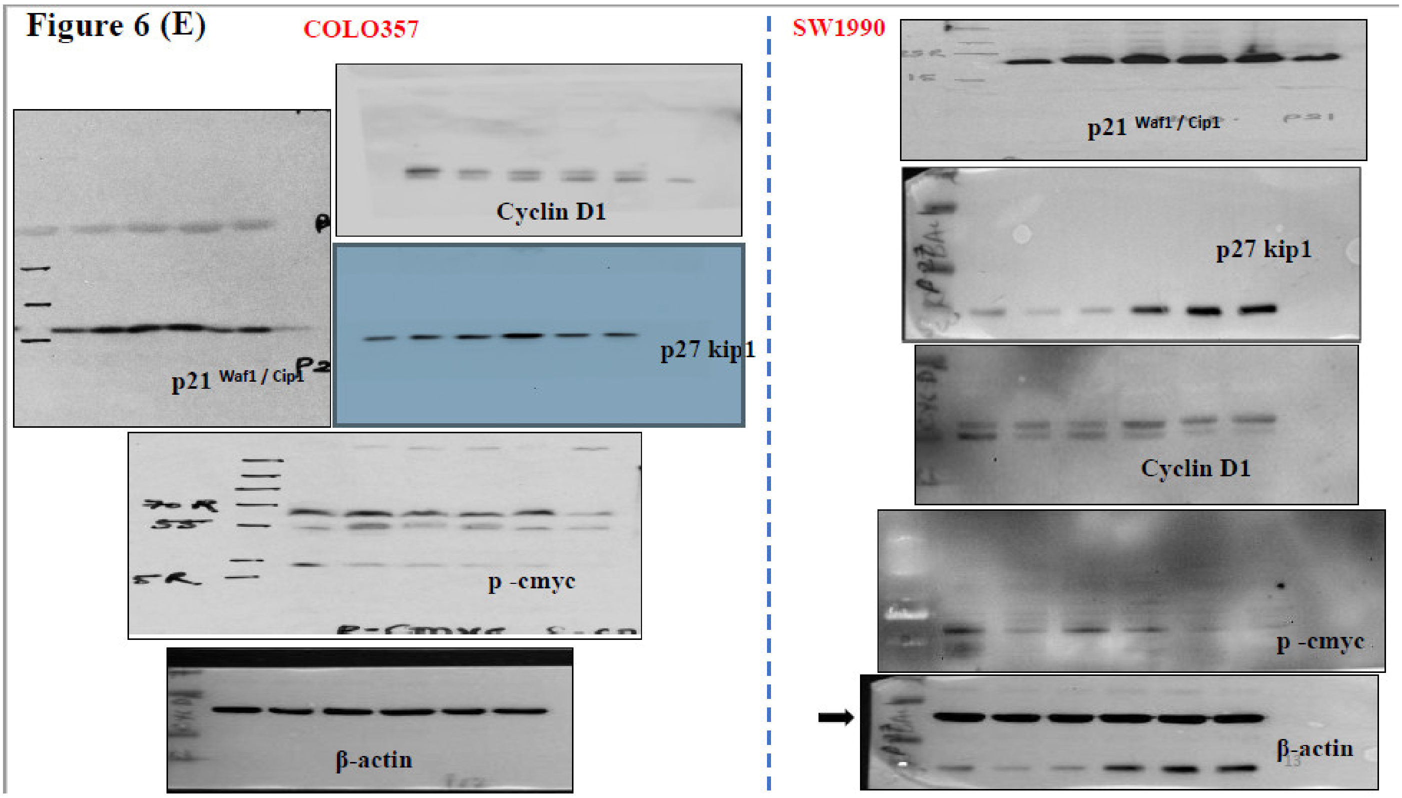

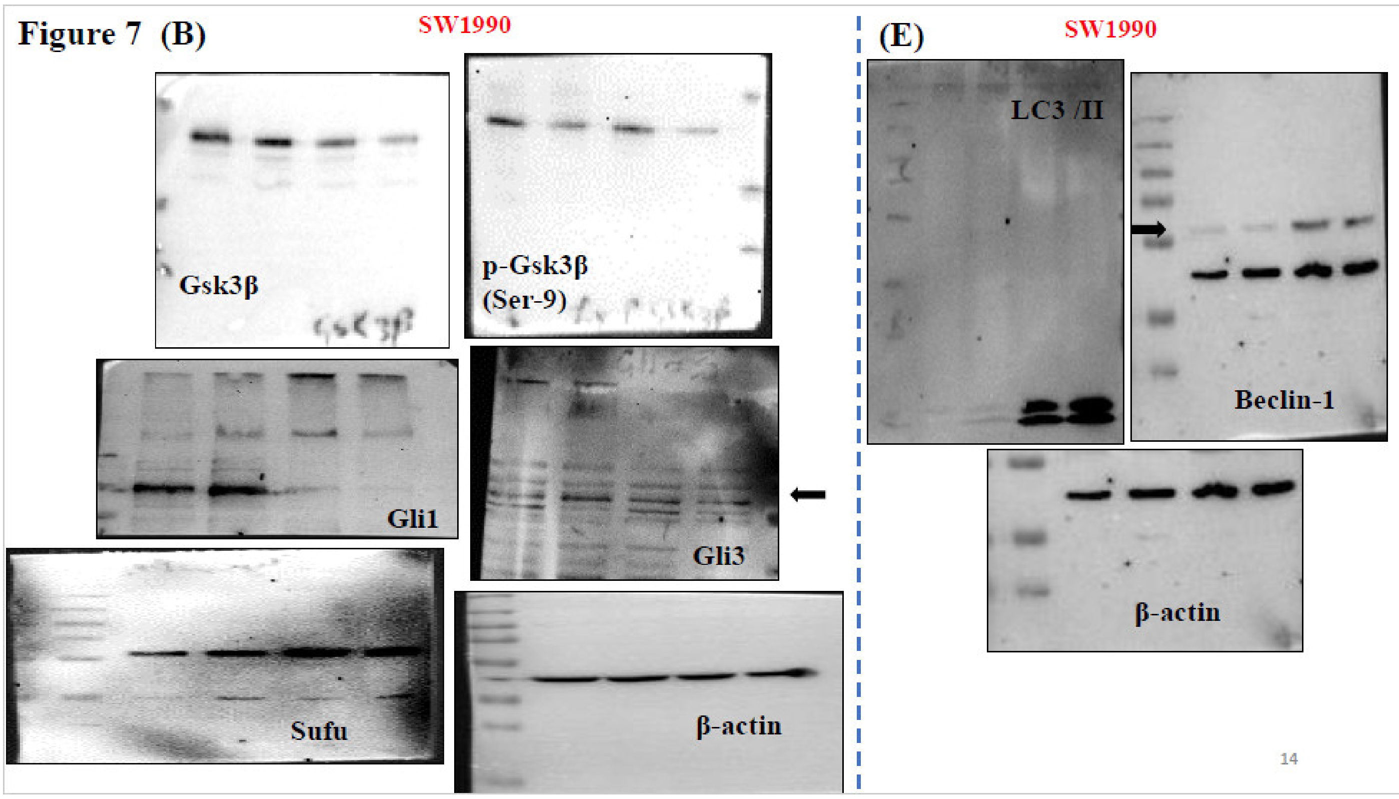

Figure S11. The whole western blot images of Figure 2, Figure 3, Figure 4, Figure 5, Figure 6, Figure 7

Table S1. List of primary antibodies.

	Primary antibody (Ab)	Cat# No.	Manufacturer	Primary Ab Dilution used	Application
1	cleaved PARP	9542	Cell Signaling Technology	1:1000	WB
2	cleaved caspase 9	20750	Cell Signaling Technology	1:1000	WB
3	LC3AI/BII	4108	Cell Signaling Technology	1:2000	WB
4	LC3	AP1802a	Abgent	1:200	IF
5	Beclin-1	sc-11427	Santa Cruz Biotechnology	1:2000	WB
6	LAMP2	PA1-655	Thermo Fisher Scientific	1:2000	WB
7	SQSTM1/p62 Antibody	5114	Cell Signaling Technology	1:1000	WB
8	BCL2	PA5-27094	Thermo Fisher Scientific	1:1000	WB
9	β-Catenin	9562	Cell Signaling Technology	1:1000	WB
10	Non-phospho (Active) β-Catenin (Ser33/37/Thr41)	8814	Cell Signaling Technology	1:1000; 1:200	WB; IF
11	p27 kip1	2552S	Cell Signaling Technology	1: 1000	WB
12	Cyclin D1	sc-8396	Santa Cruz Biotechnology	1:1000	WB
13	p21 waf1/cip1	2947S	Cell Signaling Technology	1:1000	WB
14	p- Gsk3β (Ser9)	9322	Cell Signaling Technology	1:1000; 1:100	WB; IF
15	Gsk3β	12456	Cell Signaling Technology	1:1000	WB
16	p-P70 S6K1 (Thr 421/Ser 424)	9204	Cell Signaling Technology	1:1000	WB
17	p-4EBP1(Ser-65)	9451	Santa Cruz Biotechnology	1:1000	WB
18	Gli3	19949-1-AP	Thermo Fisher Scientific	1:1000; 1:100	WB; IF
19	Gli1	sc-515781	Santa Cruz Biotechnology	1:1000; 1:100	WB; IF
20	Sufu	PA5-29952	Thermo Fisher Scientific	1:2000	WB
21	Shh	sc-373779	Santa Cruz Biotechnology	1:1000	WB
22	E-cadherin	3195	Cell Signaling Technology	1:1000	IF
23	N-cadherin	ab76011	abcam	1:1000	WB
24	Claudin-1	ab15098	abcam	1:1000	WB
25	Lamin B1	12586	Cell Signaling Technology	1:1000	WB
26	GAPDH	5174	Cell Signaling Technology	1:1000	WB
27	Cleaved Caspase-3	9664	Cell Signaling Technology	1:1000	WB
28	α-SMA	56856	Cell Signaling Technology	1:1000	WB
29	FAP-1	ab 53066	abcam	1:1000	WB
30	β-actin	sc-47778	Santa Cruz Biotechnology	1:5000	WB

Table S2. List of secondary antibodies.

S.No.	Secondary antibody (Ab)	Cat# No.	Manufacturer	Secondary Ab Dilution used
1	Goat anti-rabbit IgG-HRP	31460	Invitrogen	1:5000

2	Goat anti-mouse IgG-HRP	31430	Invitrogen	1:5000
3	Goat anti-Rabbit IgG (H+L) Secondary Antibody, Alexa Fluor 568	A-11011	Invitrogen	1:200; 1:500
4	Goat anti-Mouse IgG (H+L) Secondary Antibody, Alexa Fluor 568	A-11004	Invitrogen	1:200; 1:500
5	Goat anti-Rabbit IgG (H+L) Secondary Antibody, Alexa Fluor 488	A-11034	Invitrogen	1:200
